# Platelet-mediated progression of fatty liver disease in metabolic dysfunction-associated steatohepatitis: a pathophysiological review

**DOI:** 10.3389/fmed.2026.1728096

**Published:** 2026-04-22

**Authors:** Naif M. Alhawiti

**Affiliations:** 1 Department of Clinical Laboratory Sciences, College of Applied Medical Sciences, King Saud bin Abdulaziz University for Health Sciences, Riyadh, Saudi Arabia; 2 King Abdullah International Medical Research Center, Ministry of National Guard Health Affairs, Riyadh, Saudi Arabia

**Keywords:** liver fibrosis, liver inflammation, liver injury, MASH, MASLD, platelet therapy, platelets

## Abstract

Metabolic dysfunction-associated steatohepatitis (MASH) represents a progressive stage of metabolic dysfunction-associated steatotic liver disease (MASLD), marked by hepatic inflammation, cellular injury, and fibrosis, with potential progression to cirrhosis and hepatocellular carcinoma if not appropriately managed. Increasing evidence highlights that platelets play a significant role in MASH pathogenesis beyond their classical function in hemostasis. Enhanced platelet activation and alterations in platelet indices have been strongly correlated with disease severity, inflammatory activity, fibrotic progression, and unfavorable clinical outcomes. This review summarizes current insights into the mechanistic involvement of platelets in MASH, emphasizing their roles in inflammatory pathways, immune cell interactions, oxidative stress, and dysregulated coagulation, in addition to their interplay with metabolic disturbances. It also evaluates the utility of platelet-related markers as non-invasive indicators for disease progression and clinical monitoring. Moreover, the review explores the therapeutic potential of targeting platelet activity, including the application of antiplatelet agents either alone or in combination with antifibrotic and metabolic therapies. Although these approaches show encouraging potential, robust clinical evidence is still required to establish their safety and effectiveness. Despite recent advances, important gaps persist in understanding the molecular basis of platelet–liver interactions. Future investigations should focus on clarifying these mechanisms to facilitate the development of precise, personalized treatment strategies for MASH.

## Introduction

Metabolic Dysfunction-Associated Steatotic Liver Disease (MASLD) is a condition that affects millions of people worldwide. It is characterized by the accumulation of fat in the liver, which can cause inflammation, scarring, and liver damage over time in progressive stages (1, 2). It encompasses a spectrum of liver diseases, ranging from simple steatosis (fatty liver) to MASH, which can progress to cirrhosis and hepatocellular carcinoma (HCC). MASLD and metabolic dysfunction-associated steatohepatitis (MASH) are more recent terms currently used worldwide to best describe liver conditions highly associated with metabolic dysfunction. Recent studies have highlighted the role of platelets in the development and progression of MASH (1, 3). Patients with MASH exhibit elevated levels of circulating activated platelets compared to healthy individuals or those with simple steatosis. Increased platelet activation is associated with the severity of liver inflammation, fibrosis, and disease progression (4, 5).

Mechanisms linking platelets and MASH include inflammatory mediators, oxidative stress and coagulation dysfunctions. Activated platelets release various inflammatory mediators, such as chemokines and cytokines mainly in response to lipid toxicity, which contribute to the recruitment and activation of hepatic stellate cells (HSC), leading to liver injury and fibrosis. Platelets can also induce oxidative stress in the liver, further exacerbating hepatocellular damage and promoting the development of MASH (6, 7). The activation of coagulation cascades triggered by platelet activation may lead to the formation of fibrin clots and microvascular thrombosis, which impair liver perfusion and exacerbate liver injury. These mechanisms have major prognostic significance in the severity of MASH and the development of liver-related complications, including cirrhosis and HCC (8).

Patients with advanced MASH exhibit a hypercoagulable state marked by increased thrombin generation, elevated fibrinogen levels, and reduced activity of natural anticoagulants such as protein C and antithrombin (9, 10). Simultaneously, platelets in MASH patients are hyperreactive, evidenced by elevated mean platelet volume (MPV), increased platelet distribution width (PDW), increased expression of activation markers (e.g., P-selectin), and heightened release of pro-inflammatory mediators such as serotonin and CD40L (5, 11). These activated platelets contribute to hepatic microthrombosis, endothelial dysfunction, and fibrogenesis through interactions with Kupffer cells, neutrophils, and hepatic stellate cells (2, 6, 12). More recent studies have shown that platelet-derived microparticles and neutrophil extracellular traps (NETs) serve as amplifiers of coagulation-inflammation crosstalk in the liver microenvironment (13). Moreover, advanced fibrosis often leads to thrombocytopenia due to splenic sequestration and reduced thrombopoietin production, complicating the clinical picture and masking underlying prothrombotic tendencies (14–16).

Our current understanding of the clinical association between platelets and MASH is limited due to multiple intricacies. The mechanistic complexity involving cellular and molecular interactions, heterogeneity of MASH, and confounding factors such as obesity, insulin resistance and type 2 diabetes are not fully elucidated. Recent research suggests that targeting platelets may have therapeutic potential in MASH (7). The use of antiplatelet agents, anticoagulants, platelet-derived growth factor (PDGF) inhibitors, and platelet-derived microvesicle inhibitors aim to evaluate clinical relevance of platelet-targeted interventions in MASH treatment, potentially leading to the development of new treatment options for this condition (7, 17, 18). These potential therapeutics precisely evaluate improvements in histological, biochemical, and imaging endpoints as well as platelet-specific parameters (19, 20). In addition, combination therapies provide a more holistic approach to MASH treatment which may prove superior to single-agent approaches where the combined action of multiple drugs produces a synergistic effect leading to a greater therapeutic effect and improved disease control targeting both the platelet-mediated inflammatory pathways and the metabolic dysregulation (21, 22).

Despite substantial advances in understanding the relationship between platelets and MASH, significant gaps remain in elucidating the underlying mechanisms, platelet-immune cell interactions, and potential therapeutic strategies. This review is focusing on the association of the multifaceted role of platelets in MASH pathophysiology from scientific literature and the advancements in therapeutic strategies for both single-agent and combination therapies.

## The pathophysiology of platelets in MASH

Platelets play a critical role in liver regeneration, blood clotting and in inflammation and immune responses. In recent years, platelets have been recognized as important players in liver physiology and pathology (7, 20). Platelets are involved in restoring liver function by releasing various growth factors and cytokines that promote liver cell proliferation and differentiation (23). Platelets also interact with liver cells, such as hepatocytes and HSC, to facilitate liver regeneration (24). In MASH, platelets may contribute to the development and progression of liver disease in several ways (6).

Platelets can interact with liver cells and promote the release of pro-inflammatory cytokines and chemokines, leading to chronic liver inflammation, which can further exacerbate liver cell damage and promote the progression of liver disease (23, 25). In MASH, hepatocyte injury initiates the recruitment and activation of platelets in the liver sinusoids (26). This process is orchestrated through upregulated adhesion molecules such as P-selectin and glycoprotein Ibα (GPIbα) that facilitate platelet tethering to activated hepatic sinusoidal endothelial cells (5, 27). Once activated, platelets release a host of mediators including serotonin, PDGF, transforming growth factor-beta (TGF-β), and chemokines like CCL5, which are potent activators of HSCs, the master fibrogenic cells in the liver (28, 29). Recent studies have also shown that platelets play a role in the regulation of liver immune response. Activated platelets act as a bridge between innate and adaptive immunity (9). They enhance NET formation, promote monocyte differentiation, and foster T-cell trafficking into hepatic tissue contributing to a sustained inflammatory milieu (30). This immune-platelet crosstalk aggravates hepatic inflammation and steatosis, further accelerating hepatocellular damage (31). Another major mechanistic insight includes the interplay between coagulation and platelet activation in MASH. Hypercoagulability, evidenced by elevated thrombin generation and fibrin deposition, coexists with increased platelet reactivity, reinforcing the cycle of hepatic microthrombi formation and ischemic injury (29). Platelet-derived microparticles (PMPs) also carry microRNAs and damage-associated molecular patterns that modulate gene expression and immune signaling in liver-resident cells. Furthermore, platelet count may be a useful biomarker for predicting the severity and progression of MASLD. Lower platelet counts have been associated with more advanced stages of the disease and a higher risk of liver-related complications such as cirrhosis and hepatocellular carcinoma (13, 32, 33). Preclinical models have confirmed that pharmacologic or genetic depletion of platelets attenuates liver injury and fibrosis (34). Research shows that mice lacking GPIbα exhibit reduced hepatic inflammation, stellate cell activation, and collagen deposition (12). These findings highlight the non-hemostatic roles of platelets in driving MASH pathophysiology.

**FIGURE 1:**
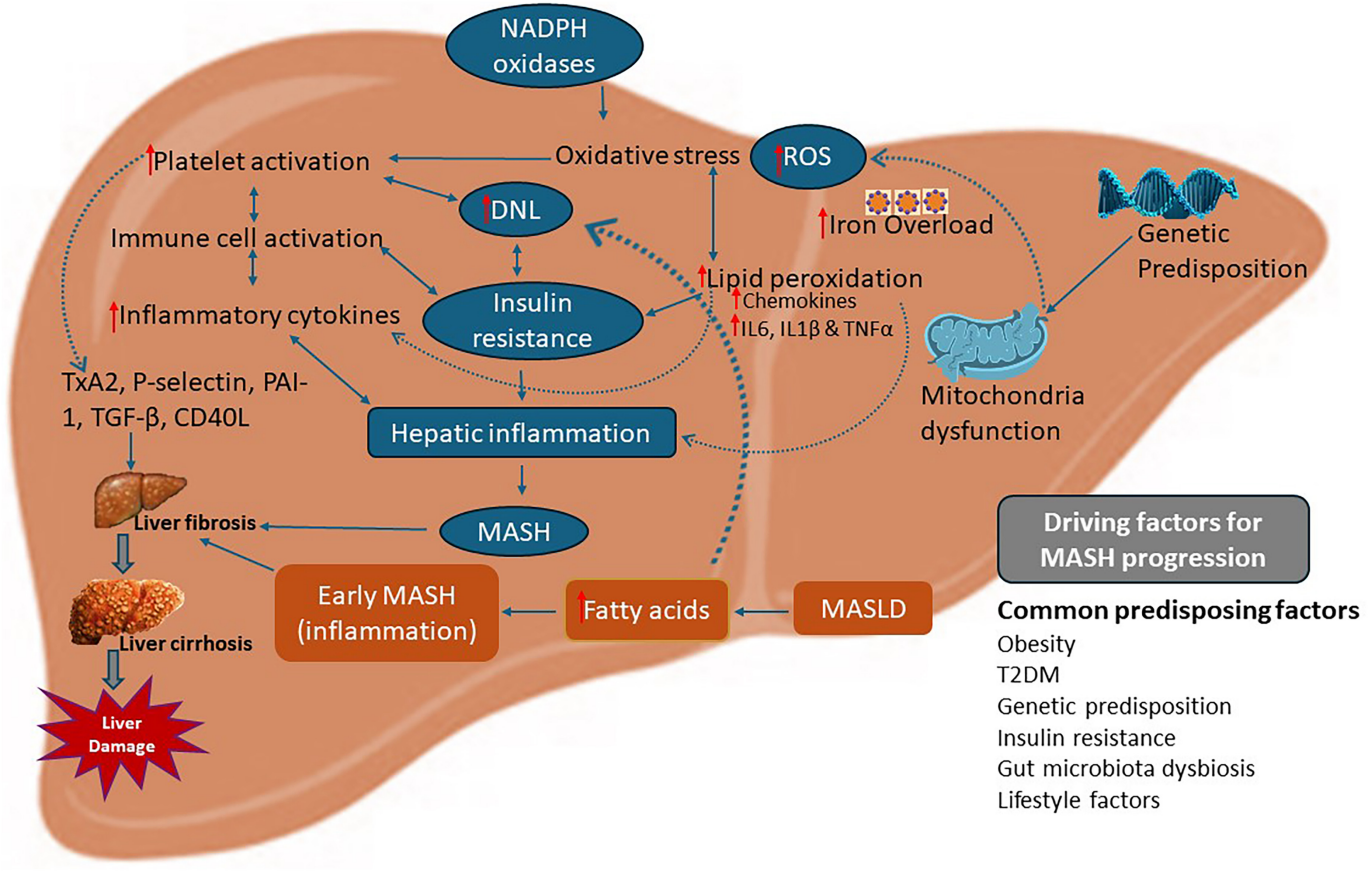
Platelet hyperactivation in the pathogenesis and progression of MASLD. Platelet activation in MASLD/MASH patients can enhance immune cell activation causing an increase in hepatic inflammatory cytokines leading to hepatic injury and exacerbation of fibrogenesis. elements such as hepatocyte damage, fibrosis, and elevated circulating free fatty acids have been included to provide a more comprehensive representation of disease progression and platelet involvement. Platelet activation has also been linked to increase lipogenesis which increases de novo lipogenesis in hepatocytes. In addition, iron overload (ferritinemia) promotes the generation of reactive oxygen species, which further amplify platelet activation, lipid peroxidation, and insulin resistance. Hepatocellular damage and inflammation are the hallmarks of MASH and cirrhosis.

Oxidative stress serves as another major pathway through which platelets influence MASH progression. Platelets may contribute to the generation of reactive oxygen species (ROS) and amplify oxidative stress in hepatocytes through NADPH oxidase activation and mitochondrial dysfunction (16). Intriguingly, another mechanism which can contribute to oxidative stress in the liver is iron overload. Iron metabolism dysregulation is increasingly implicated in the pathogenesis of MASLD and MASH. Elevated hepatic iron, particularly through hyperferritinemia, promotes oxidative stress, hepatocellular injury, and inflammation, creating a pro-thrombotic environment promoting platelet activation (35, 36). Platelet hyperreactivity, exacerbated by iron-induced oxidative stress, leads to increased secretion of pro-fibrotic cytokines and enhanced endothelial adhesion, further exacerbating liver fibrosis and progression to cirrhosis (37). Studies have shown that iron overload in MASH patients correlates with elevated MPV and platelet-derived microparticles, markers of platelet activation and vascular injury (13). Ferroptosis, a form of iron-dependent cell death, characterized by cell damage due to excessive lipid peroxidation, has also been linked to hepatocyte injury and subsequent platelet-mediated inflammation in MASH (38, 39). Therapeutic modulation of iron overload via chelation or regulation of iron-regulating proteins like hepcidin has shown potential to reduce platelet activation and alleviate liver injury (40). Thus, iron metabolism acts synergistically with platelet dysfunction to worsens lipid peroxidation and inflammation and drive the progression of MASLD as illustrated in Figure 1.

Platelets may also play a role in the development of insulin resistance, a key factor in the pathophysiology of MASLD (7). Studies have shown that platelets can release proteins such as platelet-activating factor, that stimulates de novo lipogenesis, leading to liver steatosis and further impairing insulin sensitivity (41, 42). This mechanism enhances insulin resistance and inflammation leading to the development and progression of MASH. Therefore, platelets play a complex role in the pathophysiology of MASLD (43). Their active role across the immune, oxidative, and coagulative pathways makes them powerful targets for both biomarker development and therapeutic intervention.

## Pathophysiological interaction of platelet indices with comorbidities

Platelet indices, including MPV, PDW and Plateletcrit (PCT) have emerged as important dynamic biomarkers indicative of platelet function and activation (44). Growing evidence demonstrates significant associations between these indices and systemic comorbidities, particularly diabetes mellitus (45–47) and cardiovascular diseases, highlighting their clinical significance (46–48). Metabolic disturbances, including hyperglycemia, insulin resistance, and increased oxidative stress, have been shown to enhance platelet reactivity and aggregation. These pathophysiological changes are closely associated with endothelial dysfunction, inflammatory responses, and reduced nitric oxide bioavailability, which collectively amplify platelet activation and aggregation (49, 50). As a result, cardiometabolic disorders promote platelet aggregation and hypercoagulable state, reflected by elevated MPV and PDW, indicative of larger and more reactive platelets with increased prothrombotic potential and an elevated the risk of adverse cardiovascular consequences. MASLD including MASH, which affects approximately 30% of the global population, is a major contributor to liver fibrosis and cirrhosis (51), with increasing evidence supporting platelet involvement in its pathophysiology. Accordingly, platelet indices have emerged as potential biomarkers for evaluating disease activity in MASLD/MASH and are closely linked to disease severity and progression (52).

Chronic hyperglycemia promotes non-enzymatic glycation of platelet membrane proteins and surface receptors, leading to impaired membrane fluidity and heightened platelet adhesion and aggregation (50). Hyperglycemic stress further disrupts intracellular calcium signaling and mitochondrial bioenergetics in platelets, amplifying agonist-induced activation (53). Insulin resistance constitutes a key molecular link between type 2 diabetes mellitus (T2DM) and platelet hyperreactivity; while insulin normally suppresses platelet activation via nitric oxide and prostacyclin-dependent pathways, this inhibitory signaling is attenuated in insulin-resistant states, resulting in increased surface expression of GPIIb/IIIa and P-selectin and enhanced platelet aggregation (54). These convergent metabolic and signaling abnormalities are reflected by elevations in MPV and PDW, indicative of larger, metabolically active platelets with increased prothrombotic potential (55). Notably, liver disease particularly liver cirrhosis is characterized by thrombocytopathy, which together increase the risk of pathological thrombotic events. Consequently, international guidelines recommend assessment of platelet function using laboratory techniques such as platelet aggregometry (56, 57).

T2DM accelerates the progression of MASLD, increasing the risks of liver cirrhosis, cardiovascular disease, and hepatocellular carcinoma (56). This is mediated by insulin resistance, dysregulated lipid and glucose metabolism, mitochondrial dysfunction, and oxidative stress, which collectively drive hepatic inflammation and systemic metabolic injury. MASH, the inflammatory phenotype of MASLD, frequently coexists with T2DM, amplifying liver fibrosis and cardiometabolic risk (56, 57). In MASH, chronic inflammation and cardiometabolic stress promote platelet activation and dysregulated megakaryopoiesis, contributing to immune-mediated hepatic injury. Clinically, insulin resistance–associated MASLD is characterized by reduced platelet count and PCT alongside elevated PDW, reflecting enhanced platelet activation, supporting platelet indices as mechanistic biomarkers of disease activity and progression (57). MASLD encompasses MASH, characterized by hepatocellular injury, inflammation, and liver fibrosis. Progressive adipose tissue dysfunction in systemic metabolic disorders establishes a proinflammatory and prothrombotic milieu through dysregulated adipokines, cytokines, and free fatty acids, which promote hepatic inflammation and fibrogenesis. Key mediators, including interleukin-6, tumor necrosis factor-α, and leptin, directly activate platelets and modulate megakaryocyte maturation, leading to the release of larger, more reactive platelets (58, 59). Dyslipidemia particularly elevated oxidized low-density lipoprotein cholesterol and triglycerides further enhances platelet adhesion, degranulation, and aggregation, linking increased MPV and PDW to platelet heterogeneity and vascular instability (60, 61). Together, these metabolic, inflammatory, and lipid-driven pathways interconnect hepatic injury with hemostatic dysfunction, highlighting platelet indices as mechanistic biomarkers of MASLD activity, progression, and associated cardiometabolic risk (58).

In MASLD, platelet dysfunction and dysregulated hemostasis are central contributors to disease progression and thrombotic risk (62, 63). Cardiometabolic comorbidities enhance platelet–coagulation interactions, with activated platelets providing negatively charged phospholipid surfaces that promote assembly of tenase (VIIIa–IXa) and prothrombinase (Va–Xa) complexes, intensifying thrombin generation and fibrin formation. Concurrently, increased phosphatidylserine exposure, granule secretion, and release of platelet-derived mediators such as platelet factor 4, polyphosphates, and microparticles modulate coagulation, suppress anticoagulant pathways, and stabilize thrombi. Dyslipidemia and systemic inflammation further enhance platelet activation, while endothelial injury amplifies platelet adhesion under high shear. MASLD-associated alterations in endogenous anticoagulant and fibrinolytic systems including reduced antithrombin and protein C activity and elevated plasminogen activator inhibitor-1 impair fibrinolysis (12, 64–67). These convergent pathways are reflected in increased MPV, PDW, and PCT, linking platelet hyperactivity to prothrombotic states and increased cardiovascular risk (68, 69).

Specifically, platelets are now described as dynamic regulators of liver disease with both profibrotic and antifibrotic roles. In chronic liver injury, platelets predominantly promote fibrosis through the release of profibrogenic mediators, including TGF-β and PDGF, which stimulate hepatic stellate cell activation, proliferation, and extracellular matrix deposition. In addition, platelets enhance inflammatory responses via interactions with Kupffer cells and circulating leukocytes, and contribute to sinusoidal microthrombosis, thereby exacerbating hypoxia and fibrotic remodeling (28, 29). Conversely, in acute or early-stage liver injury, platelets may exert protective and regenerative effects. They release hepatotrophic factors such as hepatocyte growth factor (HGF) and insulin-like growth factor-1 (IGF-1), which promote hepatocyte proliferation (70). Platelets may also facilitate fibrosis resolution by modulating the balance between matrix metalloproteinases and their inhibitors. Furthermore, in viral hepatitis, platelets contribute to immune cell recruitment but may also sustain chronic inflammation (16), whereas in metabolic and alcohol-related liver disease, increased platelet activation is more strongly associated with persistent inflammatory and fibrotic signaling (9). In advanced stages, such as cirrhosis, thrombocytopenia is common; however, the remaining platelets often exhibit heightened activation, continuing to influence disease progression.

## Iron-mediated dysregulation of platelet function in liver disease

The progression of chronic liver disease from MASH to cirrhosis and hepatocellular carcinoma arises from a complex interplay of metabolic dysregulation, chronic inflammation, and hemostatic disturbances (71, 72). Within this context, platelet abnormalities including altered indices and hyperreactivity emerge as critical mediators, particularly when modulated by iron-dependent regulatory pathways involving ferritin, ferroportin, and iron-sensitive signaling molecules. Platelets act as active inflammatory and immune effectors: in MASLD, metabolic stress, and lipotoxicity trigger platelet activation reflected in MPV, PDW, and PCT (73). In addition, activated platelets release pro-inflammatory and profibrogenic mediators such as platelet factor 4, transforming growth factor-β, and serotonin, driving hepatic stellate cell (HSC) activation, extracellular matrix deposition, and progression from steatosis to steatohepatitis and early fibrosis (71, 72).

Iron dysregulation serves as a mechanistic link between platelet dysfunction and liver disease progression. In MASLD, hepatic iron overload amplifies oxidative stress through ROS generation, driving lipid peroxidation, hepatocellular injury, and inflammatory signaling (13, 71). Iron-regulatory molecules, including ferritin and hepcidin, influence platelet production and activity, with altered ferritin pools reflecting disrupted iron storage and systemic inflammation, thereby promoting platelet hyperreactivity and a prothrombotic state (74). Supporting this association, Guo et al. (75) reported a positive correlation between serum ferritin concentrations, MASLD severity, and hepatic fibrosis. Elevated ferritin levels are thus associated with increased platelet size variability and reactivity, linking iron overload to abnormal platelet indices and heightened thrombotic risk.

Ferroportin is the main cellular iron exporter that regulates both intracellular and systemic iron homeostasis (73). In liver disease, its function is often disrupted by inflammatory signaling and abnormal hepcidin expression, leading to iron accumulation in hepatocytes, macrophages, and possibly megakaryocytes (71, 73, 74). Iron accumulation in megakaryocytes can impair platelet biogenesis, leading to altered platelet size, morphology, and function (75, 76). These functionally compromised platelets exacerbate sinusoidal endothelial damage and promote intrahepatic thrombosis, thereby accelerating fibrogenesis and the progression toward cirrhosis (77, 78). In MASLD, hepcidin-driven inflammation suppresses ferroportin expression, leading to hepatic iron retention (79, 80). In hepatocellular carcinoma, interactions between platelets and iron signaling pathways further promote tumor growth, immune modulation, and metastatic dissemination (81). Moreover, iron overload coupled with impaired ferritin–ferroportin signaling contributes to genomic instability and enhances resistance of cancer cells to therapy (82). Collectively, platelet hyperactivity, iron-induced oxidative stress, and chronic inflammation form interconnected pathogenic pathways that drive the progression of MASLD/MASH, liver cirrhosis, and hepatocellular carcinoma (77, 83, 84). Accordingly, iron-regulatory molecules represent promising biomarkers and therapeutic targets for chronic liver disease and liver cancer.

## Iron trafficking and regulatory proteins in MASLD

MASLD involves coordinated disturbances in lipid and iron metabolism, with iron dyshomeostasis acting as a critical amplifier of hepatocellular injury (76). Physiologically, hepatic iron trafficking is tightly regulated to balance its essential role in mitochondrial respiration, heme and iron-sulfur cluster synthesis, and DNA replication against its redox activity, which drives ROS generation through Fenton reaction (85, 86). In MASLD, disruption of systemic and intracellular iron control expands the labile iron pool, potentiating oxidative stress, lipid peroxidation, and cell apoptosis (13, 71). Clinically, approximately one-third of patients display hyperferritinemia with normal or mildly elevated transferrin saturation and modest hepatic iron deposition-features consistent with dysmetabolic iron overload. Excess ferrous iron catalyzes hydroxyl radical formation, promoting peroxidation of polyunsaturated fatty acids within steatotic hepatocytes, mitochondrial dysfunction, and ferroptotic cell death, thereby accelerating progression to MASH (85, 87).

The hepcidin-ferroportin pathway represents the principal mechanism governing systemic iron homeostasis. Hepcidin-mediated ferroportin degradation limits iron efflux from enterocytes, macrophages, and hepatocytes (79, 80). In MASLD, this hepcidin-ferroportin pathway is dysregulated: inflammatory signaling (e.g., IL-6/STAT3) may inappropriately increase hepcidin, causing hepatic iron sequestration (80, 88). Conversely, in advanced stages of disease, hepcidin levels may be inappropriately low relative to hepatic iron burden, potentially due to bone morphogenic protein (BMP)/SMAD signaling pathway or defective sensing of transferrin saturation. This relative hepcidin deficiency promotes increased intestinal iron absorption and hepatic iron accumulation, further intensifying oxidative stress and hepatocellular injury (88, 89). Intracellular iron is stored in ferritin, composed of Ferritin Heavy Chain 1 (FTH1) and Ferritin Light Chain (FTL) subunits, and ferritin upregulation reflects both iron accumulation and inflammatory signaling, explaining the frequent hyperferritinemia observed in MASLD. However, Nuclear receptor coactivator 4 (NCOA4)-mediated ferritinophagy releases iron into the labile iron pool, increasing susceptibility to ferroptosis-an iron-dependent form of lipid peroxidation-driven cell death (90). The primary genetic modifiers of iron handling causing the most common form of hereditary hemochromatosis (HFE-HH). Mutations in the HFE gene represent the principal genetic determinants of iron dysregulation underlying the most prevalent form of HFE-HH. These variants can aggravate hepatic iron deposition and potentiate disease severity when present in individuals with MASLD (91). Overall, impaired iron trafficking in MASLD is mechanistically linked to oxidative stress, ferroptotic cell death, inflammatory activation, and fibrogenic remodeling, indicating that iron overload functions as an active pathogenic contributor rather than a secondary epiphenomenon.

## Platelet’s role in liver inflammation

Inflammation is a hallmark pathological feature of MASH and the role of platelets in inflammation is complex and multifactorial. Recent findings suggest that platelets are potent drivers of hepatic inflammation beyond their widely recognized hemostatic functions (28, 92). Platelets interact dynamically with various immune cells and liver-resident cells to initiate, increase, and sustain the inflammatory environment characteristic of MASH. Platelet activation in the liver vasculature, causes their adherence to the sinusoidal endothelium via P-selectin and integrins, enabling direct interaction with kupffer cells, neutrophils, and monocytes (27, 93, 94). These interactions promote the recruitment and migration of immune cells into the hepatic parenchyma. Activated platelets trigger NET formation, discussed above, is another important mechanism which exacerbates oxidative damage and promotes further hepatocyte injury (29).

**FIGURE 2:**
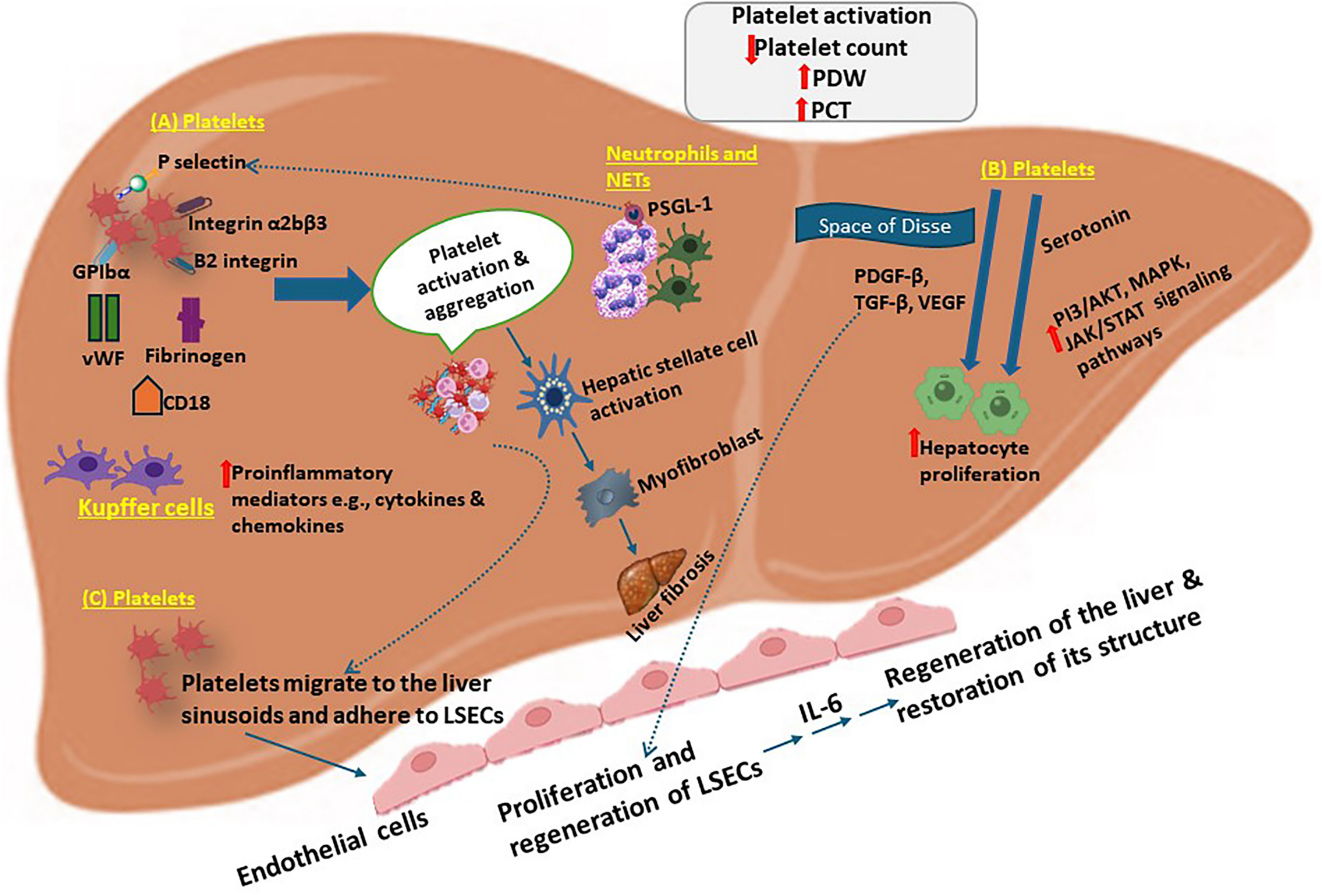
Platelet–liver cell interactions: **(A)** Platelets migrate to liver sinusoids and adhere to liver sinusoidal endothelial cells (LSECs) and Kupffer cells via GPIb–VWF and CD18-mediated interactions, triggering platelet activation, aggregation, and degranulation. These aggregates facilitate neutrophil recruitment and NET formation, while also enhancing Kupffer cell–driven inflammatory responses. **(B)** Platelets can enter the space of Disse and interact directly with hepatocytes, releasing factors such as TGF-β, PDGF, and VEGF to promote proliferation. Platelet-derived serotonin further regulates hepatocyte survival and function through PI3K/Akt, MAPK, and JAK/STAT pathways. **(C)** Following liver injury, platelets release proangiogenic factors (e.g., HGF, IGF-1, VEGF) that support LSEC regeneration. Platelet–LSEC interactions also stimulate IL-6 production, promoting hepatocyte proliferation.

Activated platelets secrete various pro-inflammatory cytokines, such as interleukin-1β, CD18, CD40 ligand, and tumor necrosis factor-alpha (TNF-α) (30), which in turn activate HSCs and endothelial cells, actively contributing to the overall inflammatory feedback loop (30, 95). Moreover, they release high mobility group box 1 and serotonin, which are known to modulate hepatic immune cell activation and promote steatohepatitis (12, 25, 96). Emerging studies also show that platelets modulate T-cell responses in MASH. Through surface molecules such as CD154, (CD40L) and MHC class I, platelets act as non-conventional antigen-presenting cells that can influence the differentiation and polarization of CD4 + and CD8 + T cells, further intensifying the adaptive immune response in the liver (25, 97, 98). Studies in mouse models of MASH have confirmed the inflammatory contribution of platelets. Genetic ablation or pharmacological inhibition of platelet receptors (e.g., GPIbα, CLEC-2) significantly reduces hepatic leukocyte infiltration, cytokine expression, and serum alanine aminotransferase levels, a marker of liver injury (5, 98). Furthermore, GPIbα knockout mice exhibit a marked reduction in hepatic macrophage accumulation and inflammatory cytokines (99, 100), highlighting the central role of platelet-endothelial interactions in MASH pathogenesis. Interestingly, platelet-derived microparticles and thromboxane A2 induce vasoconstriction impairing hepatic blood flow and oxygen delivery, further damaging hepatic tissue and promoting further immune activation (12, 101) (Figure 2).

## Platelet’s role in liver fibrosis-scoring systems

Liver fibrogenesis is another important hallmark of progressive MASH condition and is the primary determinant of liver-related morbidity and mortality (102, 103). A liver biopsy, gold standard, assesses the burden of fibrosis in clinical practice which is increasingly estimated through non-invasive scoring systems (104). Among the biomarkers integrated into these scoring algorithms, platelet count serves as a pivotal and accessible parameter, reflecting the intimate link between platelet dynamics and hepatic fibrogenesis (25, 105). Thrombocytopenia is a well-recognized surrogate for advanced liver disease. As fibrosis advances, splenic sequestration increases and thrombopoietin production decreases, leading to reduced platelet counts. (106, 107). Thus, low platelet levels correlate inversely with fibrosis stage and are included in widely used scoring systems such as the FIB-4 index, AST to Platelet Ratio Index (APRI) and the MASLD fibrosis score, both of which combine age, transaminases, BMI, and platelet count to stratify patients into risk categories (28, 105). Platelet-derived growth factors (e.g., PDGF-BB, TGF-β1) stimulate HSCs, promoting their activation into myofibroblasts, the primary producers of extracellular matrix (ECM) promoting liver fibrogenesis (108, 109). These mediators are released upon platelet degranulation, especially in the setting of chronic liver injury. Furthermore, PMPs and circulating soluble P-selectin are shed during platelet activation and carry fibrogenic cytokines and RNAs that modulate hepatic cellular responses (110). Their quantification, though currently experimental, may enhance the predictive capacity of existing fibrosis scoring systems (109).

As discussed earlier, experimental models have shown that mice lacking specific platelet receptors display reduced hepatic fibrosis when exposed to MASH-inducing diets (5, 109, 111). These findings indicate that platelet activation is not a passive biomarker but an active contributor in fibrosis progression. Clinical studies have also observed discrepancies in platelet indices such as MPV and platelet distribution width (PDW) in MASH patients, which correlate with histological fibrosis (12, 112). In conditions associated with increased platelet consumption or destruction, such as inflammation, thrombosis, or immune-mediated mechanisms, the bone marrow compensates by accelerating thrombopoiesis, resulting in the release of reticulated platelets into the circulation to restore platelet numbers. Reticulated platelets are larger than mature platelets, retain residual RNA, and exhibit increased metabolic and enzymatic activity. Their increased presence contributes to an elevated MPV. Importantly, these immature platelets are functionally more reactive, showing enhanced surface receptor expression and greater aggregatory capacity. Therefore, an increased MPV reflects not only heightened platelet turnover but also a prothrombotic and hyperreactive platelet phenotype (113, 114). Supporting this concept, Zanetto et al. demonstrated that reticulated platelets are significantly increased and hyperactivated in patients with cirrhosis, independent of disease etiology and severity, suggesting their potential utility as an additional biomarker for improving clinical management in cirrhotic patients (115).

## Biochemical and molecular interconnections in MASLD

MASLD arises from chronic, low-grade inflammation, metabolic disturbances, and hepatocellular stress. Its pathogenesis is orchestrated by complex interactions among hepatocytes, non-parenchymal liver cells, and circulating mediators, including growth factors, cytokines, and chemokines. Lipotoxic injury to hepatocytes activates immune cells-particularly Kupffer cells and T lymphocytes which secrete pro-inflammatory cytokines such as tumor necrosis factor-α (TNF-α), IL-6, and interleukin-1β (IL-1β), amplifying hepatocyte stress, apoptosis, and liver injury. Concurrently, chemokines including CCL2 and CXCL10 recruit monocytes and other immune cells, further sustaining the inflammatory cascade. Platelets play a pivotal role in MASLD progression through the release of diverse bioactive mediators. PDGF and TGF-β activate hepatic stellate cells, promoting extracellular matrix deposition and fibrogenesis. VEGF and serotonin influence sinusoidal endothelial cell function, vascular tone, and microcirculatory remodeling, while platelet chemokines such as CXCL4 and CCL5 recruit immune cells and amplify local inflammation. These platelet-derived signals synergize with cytokines and chemokines from immune cells and adipose tissue, linking metabolic dysfunction to hepatocyte injury, inflammation, and fibrosis. Together, this intricate network of platelet- and immune-mediated signals highlights the multifactorial biochemical and molecular crosstalk driving MASLD progression and identifies potential therapeutic targets for modulating inflammation, fibrogenesis, and metabolic stress. In this review, the roles of three key growth factors-PDGF, TGF-β, and VEGF-are discussed in larger details.

### Platelet-derived growth factor

Platelet-derived growth factor (PDGF) is a dimeric glycoprotein comprising four isoforms-PDGF-A, PDGF-B, PDGF-C, and PDGF-D-and functions as a highly potent mitogenic and chemotactic regulator of HSC activation in MASLD/MASH (116). Upon platelet degranulation within the inflamed hepatic microenvironment, particularly through release of the PDGF-BB isoform, PDGF ligands engage PDGF receptors (PDGFR-α and PDGFR-β), which are markedly upregulated on activated HSCs. Ligand binding promotes receptor dimerization and autophosphorylation at specific intracellular tyrosine residues, thereby creating recruitment sites for adaptor molecules that trigger diverse downstream signaling networks. Key pathways include PI3K/Akt, which supports HSC survival and resistance to apoptosis; the Ras/Raf/MEK/ERK (MAPK) cascade, which mediates proliferation and migration; and JAK/STAT signaling, which enhances transcription of profibrotic genes such as collagen type I and TIMP-1 (105). Interaction of PDGF-B and PDGF-D with PDGFR-β further augments ERK/MAPK and PI3K/Akt (PKB) activation, intensifying proliferative responses. Concurrently, diminished hepatic phosphoenolpyruvate carboxykinase 1 (PCK1) expression promotes PDGF-A secretion, facilitating MASLD progression via the RhoA/PI3K/Akt axis (117, 118) (Figure 2).

In obesity-associated type 2 diabetes, CpG hypomethylation within the PDGFA gene drives its overexpression, thereby worsening hepatic insulin resistance and disease advancement (118). PDGF additionally promotes HSC activation and fibrogenesis through SHP2–REDD1–mTOR–dependent extracellular vesicle release. Notably, PDGF signaling interacts synergistically with TGF-β/SMAD pathways to amplify extracellular matrix accumulation and myofibroblastic transdifferentiation. Oxidative stress arising from lipotoxicity, mitochondrial dysfunction, and iron overload further enhances PDGFR activity by sustaining receptor phosphorylation and prolonging downstream kinase signaling (119, 120). Furthermore, thrombin-induced PAR-1 activation and platelet-derived microparticles significantly potentiate PDGF-mediated signaling in liver fibrosis, linking coagulation and inflammatory pathways with stellate cell activation. Insulin resistance and compensatory hyperinsulinemia may further increase HSC responsiveness to PDGF through upregulation of PDGFR expression and modulation of intracellular metabolic signaling (118, 120). Collectively, these interrelated molecular mechanisms establish PDGF signaling as a central integrative hub connecting platelet activation, metabolic imbalance, oxidative stress, and coagulation abnormalities to progressive hepatic fibrotic remodeling in MASLD/MASH.

### Transforming growth factor-β

Transforming growth factor-beta (TGF-β) is a pivotal cytokine secreted by multiple hepatic cell types that mediate HSC activation and drives hepatic fibrogenesis. The intracellular transcriptional mediators of this pathway, the small mothers against decapentaplegic proteins (SMADs), serve as crucial effectors of TGF-β signaling (121). In the recognized TGF-β/SMAD cascade, TGF-β1 engages the type I TGF-β receptor kinase, triggering phosphorylation of SMAD2 and SMAD3, which subsequently translocate to the nucleus to regulate transcription of profibrotic target genes, including Col1a1 and Pai1 (122). TGF-β1 has been implicated in the progression of advanced fibrosis in MASH, with the TGF-β1/pSMAD2/SP3-M1 axis inducing oxidative DNA damage in activated HSCs from affected patients (123). Moreover, interplay between peroxisome proliferator-activated receptor gamma (PPAR-γ) and TGF-β1/SMAD signaling amplifies HSC activation, hepatic inflammation, and fibrotic remodeling, as observed in methionine and choline-deficient (MCD) diet models (124). Cooperatively, TGF-β signaling, in coordination with other molecular pathways, acts synergistically to exacerbate MASLD progression and liver fibrosis.

### Vascular endothelial growth factor

Vascular endothelial growth factor (VEGF), primarily secreted by liver sinusoidal endothelial cells (LSECs) and hepatic stellate cells (HSCs), exerts context-dependent effects on liver fibrosis via VEGF receptors (VEGFRs) (117). During liver fibrosis development, VEGF promotes inflammation, HSC proliferation, and angiogenesis, whereas VEGFR2 or VEGF-B inhibition attenuates steatosis, inflammation, and MASLD progression through targeting lipolysis in white adipose tissue (125). Conversely, myeloid cell–derived VEGF can facilitate fibrosis regression by enhancing extracellular matrix degradation, increasing sinusoidal permeability, and recruiting scar-associated macrophages (SAMs) that mediate antifibrotic responses through the antifibrotic chemokine CXCL9 and metalloproteinase 13 (MMP13) (126, 127). Furthermore, TGF-β, PDGF, and VEGF are central drivers of HSC activation, supplemented by pathways such as connective tissue growth factor (CTGF), Hedgehog, and Notch signaling (23). As these profibrotic mediators originate mainly from LSECs and inflammatory cells, HSC activation largely reflects secondary responses to angiocrine and immune cues. Therapeutic strategies should therefore focus on modulating LSEC and inflammatory cell activity to preserve sinusoidal homeostasis and limit fibrotic progression (128–130).

## Platelets as therapeutic targets for MASH

The recognition of platelets as active contributors to hepatic inflammation and fibrosis in MASH has prompted large interest in their potential as therapeutic targets (12, 131, 132). Platelet-mediated signaling affects key pathogenic pathways in MASH as discussed earlier, including immune cell recruitment, HSC activation, and oxidative cell injury. Thus, modulating platelet activity may offer novel disease-modifying strategies (127–130). Antiplatelet agents have emerged as promising therapeutic candidates. Several preclinical studies have demonstrated that pharmacological inhibition of platelet function mitigates liver injury and fibrosis (5, 133). In mouse models of MASH, treatment with aspirin or clopidogrel, a P2Y12 receptor antagonist, resulted in decreased serum ALT levels, reduced hepatic infiltration of immune cells, and lower expression of profibrotic markers such as α-SMA and collagen type I (134). These findings underscore the importance of inhibiting platelet activation to halt/ treat hepatic damage in MASH patients. Beyond conventional agents, targeting specific platelet receptors and ligands involved in hepatic accumulation presents an attractive novel strategy. Targeting the blockade or genetic deletion of GPIbα reduces platelet accumulation in the liver and significantly attenuates inflammation and fibrosis in MASH models (5, 110). Similarly, inhibition of C-type lectin-like receptor 2 (CLEC-2), another platelet receptor, has shown hepatoprotective effects in experimental settings (135).

In addition to conventional antiplatelet approaches, novel single-agent therapies are being developed to target platelet-derived mediators. Agents that inhibit thromboxane A2 production or block serotonin receptors have shown efficacy in reducing liver fibrosis and remodeling in preclinical studies (12, 133). Likewise, statins, widely used for dyslipidemia, possess secondary antiplatelet properties, and may indirectly limit platelet-mediated liver injury (25). Although clinical evidence is still limited, observational studies indicate that long-term low-dose aspirin use is associated with a lower risk of fibrosis progression in MASLD patients. Moreover, combination therapies that pair antiplatelet drugs with anti-inflammatory agents are currently under evaluation in early-phase clinical trials (136, 137). Another promising strategy involves the use of antibodies against platelet-derived growth factor (PDGF). Given that PDGF-particularly the PDGF-BB isoform-promotes hepatic stellate cell proliferation and collagen deposition, anti-PDGF antibodies can directly attenuate fibrotic remodeling in the liver (138). Preclinical studies in diet-induced MASH models have demonstrated that monoclonal PDGF antibodies reduce both liver fibrosis and inflammation (139). While no anti-PDGF therapies have yet been approved for MASH, investigational agents such as olaratumab (anti-PDGFR-α) are being explored in fibrotic disease settings. These approaches offer targeted antifibrotic potential, though challenges including drug delivery, immunogenicity, and cost must be addressed. Further clinical studies are needed to establish their safety and therapeutic efficacy in MASH patients.

Novel therapeutic single-agents are also being developed to interfere with platelet-derived mediators. For instance, drugs that inhibit thromboxane A2 synthesis or antagonize serotonin receptors have demonstrated efficacy in reducing fibrotic remodeling in liver tissues (12, 133). Additionally, statins, commonly used for hyperlipidemia, have shown ancillary antiplatelet effects and may indirectly suppress platelet-mediated liver damage (25). Clinical data, while still emerging, support these findings. Observational studies suggest that chronic low-dose aspirin use is associated with reduced risk of fibrosis progression in MASLD patients (136, 137). Furthermore, combination therapies involving antiplatelet agents and anti-inflammatory drugs are being evaluated in early-phase clinical trials. Another potential therapeutic strategy for targeting platelets in MASH is the use of anti-platelet-derived growth factor antibodies. As PDGF, particularly the PDGF-BB isoform, can promote liver fibrosis, anti-PDGF antibodies inhibit HSC proliferation and collagen synthesis, directly attenuating fibrotic remodeling in the liver (110). Preclinical models have demonstrated that monoclonal antibodies against PDGF reduce liver fibrosis and inflammation in diet-induced MASH mice (139). While no anti-PDGF antibodies have yet been approved specifically for MASH, agents such as olaratumab (anti-PDGFR-α) and other investigational compounds are under assessment in fibrotic disease contexts. The therapeutic promise lies in their anti-fibrotic specificity, though challenges include delivery, immunogenicity, and cost. Further trials are necessary to validate their safety and efficacy in MASH patients.

### Single-agent therapies

Several clinical trials are currently evaluating platelet-targeted interventions as potential therapies for MASH. Antiplatelet agents, including low-dose aspirin and clopidogrel, are being investigated in phase 2 studies, while anticoagulant therapies such as apixaban is being assessed for its efficacy and safety in patients with MASH (140–142). In addition, platelet-derived growth factor (PDGF) signaling is being targeted using tyrosine kinase inhibitors such as imatinib mesylate, which is also in phase 2 clinical development (143). Dipyridamole, an antiplatelet agent that inhibits platelet activation and the formation of platelet-derived microvesicles, is likewise being assessed in phase 2 trials (144). Among these agents, aspirin is the most extensively studied. Through irreversible inhibition of cyclooxygenase-1 and suppression of thromboxane A2 production, aspirin reduces platelet aggregation. In preclinical MASH models, aspirin treatment attenuated hepatic inflammation, macrophage infiltration, and fibrotic marker expression (93). Consistent with these findings, observational studies suggest that long-term low-dose aspirin use is associated with reduced fibrosis and slower progression to cirrhosis in patients with MASLD (107). Clopidogrel, a P2Y12 receptor antagonist, inhibits ADP-dependent platelet activation and platelet–immune cell interactions. In murine MASH models, clopidogrel reduced hepatic necroinflammation and fibrosis, indicating hepatoprotective effects beyond its established cardiovascular benefits (30, 145).

Statins, although primarily prescribed for dyslipidemia, exhibit indirect antiplatelet and anti-inflammatory effects. Their use has been associated with improvements in liver enzymes, hepatic histology, and inflammatory cytokine profiles in MASH patients (146, 147). Mechanistically, statins inhibit isoprenoid synthesis required for platelet activation signaling, supporting their potential role in platelet-modulating strategies. Targeted biologic approaches are also emerging, exemplified by inhibition of the platelet adhesion receptor GPIbα. Genetic deletion or pharmacological blockade of GPIbα significantly reduced hepatic inflammation and fibrosis in MASH-prone mice, highlighting this pathway as a highly specific single-agent therapeutic target (5). Other single agents target platelet-derived cytokines and chemokines. Serotonin receptor antagonists, such as ketanserin, are being explored for their ability to counteract serotonin-mediated stellate cell activation (148). Additionally, thromboxane synthase inhibitors have shown antifibrotic activity in MASH animal models (149, 150), though human data are still limited. Heterogeneity and interindividual variability in platelet responsiveness and comorbid cardiovascular conditions must be accounted for when deploying antiplatelet monotherapy. Furthermore, enoxaparin, a low–molecular-weight heparin, is clinically used to prevent portal vein thrombosis in patients with liver cirrhosis. As reported by Villa et al., enoxaparin treatment as an antithrombotic strategy was associated with delayed onset of hepatic complications and improved survival in a small randomized controlled trial; however, these findings warrant further investigation (151).

### Combination therapies

Combination therapies that integrate platelet-targeted interventions with agents addressing complementary pathogenic mechanisms are increasingly explored as a holistic approach to MASH treatment, aiming to improve efficacy beyond single-agent strategies (152). By simultaneously targeting platelet activation, inflammation, and fibrogenesis, these approaches seek to exploit synergistic effects across multiple disease pathways, leading to greater improvements in liver histology, biochemical markers, and clinical outcomes (45, 153). Compared with monotherapies, combination regimens offer several advantages, including enhanced therapeutic efficacy through multi-pathway modulation, improved disease control, and the potential to overcome resistance to single-agent treatments (153–155). In addition, combining platelet-directed therapies with immunomodulatory or metabolic agents may augment anti-inflammatory and immune-regulatory responses, further improving treatment outcomes (156). Several combination strategies have shown promise in preclinical and early clinical studies. Antiplatelet agents such as aspirin or clopidogrel, when combined with antifibrotic therapies including selonsertib or lanifibranor, synergistically reduce hepatic inflammation and fibrosis by inhibiting platelet activation and hepatic stellate cell signaling, respectively (157). Similarly, anticoagulant–antifibrotic combinations, such as apixaban with antifibrotic agents, target both coagulation abnormalities and fibrogenic pathways implicated in MASH progression (135).

Other approaches include combining antiplatelet therapy with antioxidants or metabolic modulators. Dual treatment with aspirin and vitamin E has been shown to reduce oxidative stress, hepatic inflammation, and steatosis more effectively than either agent alone in preclinical models (12, 158). Likewise, clopidogrel combined with peroxisome proliferator-activated receptor (PPAR) agonists, such as pioglitazone, improves hepatic lipid metabolism while suppressing platelet-driven and immune-mediated inflammation, resulting in greater attenuation of fibrosis markers compared with monotherapy (45, 150, 159). Combinations of statins with angiotensin receptor blockers, such as losartan, further demonstrate complementary effects by reducing platelet activity, inflammation, and angiotensin II–mediated stellate cell activation, with clinical studies reporting greater reductions in liver stiffness and aminotransferase levels (138, 160, 161). Finally, more targeted molecular strategies are emerging, including the combination of GPIbα antagonists with anti-TNF biologics, which aim to suppress both platelet-mediated hepatic recruitment and pro-inflammatory cytokine signaling. In murine models of fibrotic MASH, this approach resulted in additive reductions in leukocyte infiltration and fibrotic remodeling (5, 162). A summary of platelet-targeted therapeutic strategies in MASH is provided in Table 1.

**TABLE 1:** Single-agent and combined therapeutics in NASH/MASH targeting platelet pathways.

Therapeutic agent	Function	Primary target	Availability/trials	References
Aspirin	Antiplatelet, anti-inflammatory	COX-1, thromboxane A2	Approved (off-label in NASH)	Dalbeni et al. (4); Nowak et al. (107)
Clopidogrel	Antiplatelet via P2Y12 inhibition	P2Y12 receptor	Approved (off-label in NASH)	Dalbeni et al. (4); Hilscher and Shah (30)
Statins	Lipid-lowering, anti-inflammatory	HMG-CoA reductase, isoprenoid pathway	Approved (under study for NASH)	Sumida and Yoneda (106); Ramadori et al. (25)
GPIbα Antagonists	Inhibits platelet adhesion to sinusoidal endothelium	GPIbα on platelets	Preclinical (mouse models)	Malehmir et al. (5)
Serotonin Receptor Antagonists	Blocks serotonin-mediated stellate cell activation	5-HT receptors on stellate cells	Preclinical (experimental use)	Ogresta et al. (12)
Thromboxane Synthase Inhibitors	Inhibit thromboxane A2 and reduce vasoconstriction	Thromboxane synthase	Preclinical (limited studies)	Ogresta et al. (12)
Aspirin + Vitamin E	Antiplatelet + antioxidant/anti-inflammatory	Oxidative stress and platelet activation	Preclinical (synergy shown)	Ogresta et al. (12); Dalbeni et al. (4)
Clopidogrel + Pioglitazone	Antiplatelet + insulin sensitizer	Platelet activation + insulin resistance	Preclinical (NASH models)	Dalbeni et al. (4)
Statins + ARBs	Lipid-lowering + antifibrotic via RAAS inhibition	Platelet activation + Angiotensin II pathway	Small clinical trials ongoing	Ramadori et al. (25)
GPIbα Antagonists + Anti-TNF	Anti-adhesive + inflammation reduction	Platelet-endothelium binding + TNF	Preclinical (additive effects in mice)	Malehmir et al. (5)
GLP-1 RA + Antiplatelet	Metabolic regulation + platelet inhibition	GLP-1 receptor + platelet inflammatory pathways	Under clinical evaluation	Yin et al. (43)
Selonsertib + Antiplatelet	Apoptosis signal inhibitor + antiplatelet	ASK1 inhibition + platelet activity	Phase II/III trials (mixed results)	Hilscher and Shah (30); Friedman et al. (157)
Lanifibranor + Antiplatelet	Pan-PPAR agonist + antiplatelet	PPARα/γ/δ + platelet inflammation	Ongoing trials (e.g., NATIVE study)	Francque et al. (19); Dalbeni et al. (4)

NASH, Non-Alcoholic Steatohepatitis; MASH, Metabolic dysfunction-Associated Steatohepatitis; COX-1, Cyclooxygenase-1; P2Y12, Purinergic Receptor P2Y, G-Protein Coupled, 12; HMG-CoA, 3-Hydroxy-3-Methyl-Glutaryl-Coenzyme A; GPIbα, Glycoprotein Ib alpha; 5-HT, 5-Hydroxytryptamine; RAAS, Renin-Angiotensin-Aldosterone System; TNF, Tumor Necrosis Factor; GLP-1 RA, Glucagon-Like Peptide-1 Receptor Agonist; ARBs, Angiotensin II Receptor Blockers; MPV, Mean Platelet Volume; ASK1, Apoptosis Signal-regulating Kinase 1; PPAR, Peroxisome Proliferator-Activated Receptor.

## Bleeding risk in platelet-modulating treatments

While platelet-targeted therapies hold promise for the treatment of MASH, their potential to increase bleeding risk requires careful consideration, particularly in patients with advanced fibrosis or cirrhosis who often exhibit coagulopathy (140, 141). This concern is especially relevant for strategies that profoundly impair platelet adhesion or activation, such as genetic deletion or strong inhibition of the platelet GPIb-IX-V complex (163, 164). GP1bα is essential for platelet binding to von Willebrand factor under high shear conditions, and its loss produces a phenotype resembling Bernard–Soulier syndrome, a rare inherited bleeding disorder characterized by macrothrombocytopenia and severe hemorrhage. Pre-clinical studies demonstrate that mice lacking *Gp1bα* show markedly prolonged bleeding times, impaired hemostasis after vascular injury, and spontaneous bleeding events, despite only moderate reductions in platelet counts. These findings indicate that defective platelet adhesion, rather than thrombocytopenia alone, is the main driver of bleeding in these models (164).

Although GP1bα blockade or deletion effectively reduces platelet accumulation and fibrosis in experimental MASH, the associated hemorrhagic liability raises significant translational concerns, particularly in patients with fragile hemostatic balance (165). In contrast, conventional antiplatelet agents such as aspirin or P2Y12 inhibitors generally show a more favorable bleeding profile in pre-clinical models, though most studies are short-term and conducted in animals without pre-existing coagulopathy. These observations highlight the need for **c**areful risk benefit evaluation and tailored therapeutic strategies. As a result, precision approaches are being explored that selectively modulate platelet signaling while sparing systemic hemostasis. Potential strategies include targeting platelet-derived microvesicles, soluble adhesion molecules, or specific cytokine release, offering the prospect of highly targeted treatments that limit pathological platelet activity in MASH with minimal bleeding risk (166).

## Toxicity and safety considerations of combination therapies

Although combination therapies targeting multiple pathogenic mechanisms in MASH-such as the concomitant use of antiplatelet agents with anti-inflammatory or antifibrotic drugs may enhance therapeutic efficacy, they also introduce important concerns related to cumulative toxicity (167, 168). The concurrent administration of multiple agents can intensify adverse effects, including bleeding complications, hepatotoxicity, and systemic immune disturbances. Combining antiplatelet drugs with other therapies that influence platelet function or vascular homeostasis may increase hemorrhagic risk, especially in patients with advanced fibrosis or cirrhosis who already exhibit impaired coagulation and unstable hemostatic balance.

Moreover, therapeutic regimens involving agents with shared hepatic metabolic pathways or overlapping off-target actions may further elevate the risk of liver injury or systemic toxicity (169, 170). Clinically, the risk of cumulative toxicity is particularly relevant in elderly or comorbid populations, who are more vulnerable to gastrointestinal bleeding, thrombocytopenia, and clinically significant drug–drug interactions (7, 171). Therefore, careful selection of drug doses, systematic monitoring of safety biomarkers, and a stepwise approach in clinical trials are essential to balance therapeutic efficacy with the risk of additive or synergistic adverse effects. In summary, although combination therapies hold promise for targeting multiple pathogenic pathways in MASH, precise choice of agents, optimized dosing, and appropriate patient stratification are critical to minimize cumulative toxicity and ensure a favorable risk–benefit profile.

## Conclusion

In conclusion, the emerging understanding of platelets as multifaceted contributors to the pathogenesis of non-alcoholic steatohepatitis extends beyond their traditional role in coagulation homeostasis. Future research should focus on elucidating the precise molecular mechanisms by which platelets influence hepatic inflammation, immune cell recruitment, and fibrotic remodeling. Moreover, their potential as non-invasive biomarkers in fibrosis assessment and disease monitoring warrants validation in large-scale longitudinal studies. The therapeutic promise of antiplatelet agents-whether as standalone treatments or in combination with existing antifibrotic or metabolic therapies-requires rigorous clinical evaluation to determine optimal efficacy and safety profiles. As our understanding of platelet-liver crosstalk continues to evolve, targeted modulation of platelet activity may emerge as a cornerstone in the development of personalized strategies for MASH management and prevention.
